# Spermine Significantly Increases the Transfection Efficiency of Cationic Polymeric Gene Vectors

**DOI:** 10.3390/pharmaceutics17010131

**Published:** 2025-01-17

**Authors:** Yue Lv, Jiaoqin Xue, Pengfei Cui, Lin Qiu

**Affiliations:** School of Pharmacy, Changzhou University, Changzhou 213164, China; wanghh9913@163.com (Y.L.); 18651989237@163.com (J.X.)

**Keywords:** polyethyleneimine, spermine, transfection efficiency, endosome escape

## Abstract

**Background/Objectives:** Non-viral vectors have gained recognition for their ability to enhance the safety of gene delivery processes. Among these, polyethyleneimine (PEI) stands out as the most widely utilized cationic polymer due to its accessibility. Traditional methods of modifying PEI, such as ligand conjugation, chemical derivatization, and cross-linking, are associated with intricate preparation procedures, limited transfection efficiency, and suboptimal biocompatibility. **Methods:** In this investigation, enhanced transfection efficiency was achieved through the straightforward physical blending of PEI carriers with spermine. **Results:** Transfection assays explored the maximal enhancement potential conferred by spermine, alongside further methodological refinements aimed at optimizing transfection efficacy, showcasing a potential increase of up to 40.7%. Through the comparison of different addition sequences of spermine, the optimal complex PEI/Spermine/DNA for transfection efficiency was selected. Characterization of PEI/Spermine/DNA revealed that, compared to PEI/DNA, its particle size increased to approximately 150 nm. Molecular dynamics simulation results revealed that spermine can enhance the interaction between PEI and DNA, thereby forming a system with lower energy and greater stability. Mechanistic inquiries studies also disclosed that spermine augments the endosomal escape capability of PEI carriers without altering pathways involved in the cellular uptake of gene nanoparticles, thereby facilitating heightened gene expression. **Conclusions:** PEI-Sper emerges as a promising non-viral vector for gene delivery, distinguished by its simplicity in preparation, cost-effectiveness, and superior transfection efficiency.

## 1. Introduction

Gene therapy has arisen as a revolutionary approach for addressing rare ailments. It entails the introduction of functional genes into individuals to amplify the expression of specific proteins. Additionally, gene therapy can combat illnesses by inhibiting or diminishing the activity of target genes through the utilization of antisense technology or small interfering RNA (siRNA) [1,2]. While gene therapy holds great promise for rectifying genetic flaws responsible for hereditary disorders, its effectiveness hinges on efficient and safe delivery mechanisms [3]. Viral vectors, despite their effectiveness in transfection, present challenges due to their heightened immunogenicity and teratogenic potential [4,5]. Conversely, non-viral vectors are favored for clinical applications due to their reduced immunogenicity, minimal toxicity, precise targeting, simple preparation methods, and capacity to carry genes of various sizes [6]. Nonetheless, non-viral vectors generally display inferior transfection efficiency compared to viral counterparts, prompting ongoing research into enhancing their delivery capabilities.

Non-viral vectors encompass cationic lipids [7], cationic polymers [8,9], peptide proteins [10], and nanomaterials [11,12]. Typically, these vectors possess cationic characteristics that electrostatically interact with negatively charged nucleic acids to facilitate gene delivery. Polyethylenimine (PEI) is a cationic polymer commonly employed as a non-viral gene carrier for delivering nucleic acids. Its ability to compact deoxyribonucleic acid (DNA) into polyplexes, combined with its robust buffering capacity within endosomes, triggers osmotic pressure elevation and lysosomal rupture by inducing chloride ion influx into lysosomes [13]. This process effectively releases gene material into the cytoplasm for functionality. PEI exists in high- and low-molecular-weight forms: high-molecular-weight PEI offers superior transfection efficiency but comes with considerable toxicity, whereas low-molecular-weight PEI boasts improved biocompatibility albeit with reduced transfection efficiency. Various strategies have been developed to modify PEI [14,15,16], aiming to enhance its transfection capabilities or mitigate its adverse effects. By harnessing PEI’s numerous protonated amino groups, a variety of materials such as folic acid [17], polyethylene glycol (PEG) [18,19], amino acid derivatives [20], biomimetic structures [21,22] like cell membranes, extracellular vesicles, and biodegradable cross-linkers [23,24] like disulfide bonds have been explored to enhance PEI’s abilities and reduce its toxicity. Despite these enhancements in PEI’s functionalities, challenges persist, including complex preparation protocols and limited enhancements in transfection efficiency.

Spermine, a natural polyamine prevalent in eukaryotic cells, exhibits outstanding biocompatibility. At suitable concentrations, spermine can condense genes to enhance their stability [25,26,27]. Though spermine, as a small molecule, cannot directly transport large molecules like DNA or RNA into cells, increasing its molecular weight or collaborating with larger molecules facilitates gene delivery. Spermine’s cationic attributes also play a crucial role in augmenting gene delivery efficacy, enabling it to co-deliver genes alongside carbohydrates or proteinaceous materials with minimal biosafety concerns [28,29].

Generally speaking, the utilization of spermine in carrier modification for improving transfection techniques typically entails either covalent conjugation or association with large molecules. Nevertheless, this strategy is fundamentally marked by the inevitable downsides of complex preparation procedures and challenging acquisition.

In this investigation, PEI possessing a molecular weight of 25 kDa was combined with spermine in a precise proportion, resulting in the formulation known as PEI-Spermine through physical amalgamation. This composite serves to augment the proton-buffering capability of PEI, thereby amplifying its capacity to evade endosomal entrapment, thus fostering efficient conveyance of genetic material into the cellular nucleus for subsequent expression. The selection of plasmid DNA (pDNA) encoding green fluorescent protein (GFP) as the model gene substance for in vitro investigations allowed for the exploration of the improved transfection efficiency mechanism. This investigation involved analyzing the particle size, morphological characteristics of gene complexes, intermolecular interactions, uptake, and transfection mechanisms and observing nanoparticle (NP) distribution within cells post-uptake.

## 2. Materials and Methods

### 2.1. Materials

Polyethyleneimine (PEI, branched, molecular weight = 25 kDa, Product Number Aldrich—408727) and Salmon sperm DNA (D3159) were purchased from Sigma-Aldrich (Shanghai, China). Spermine (Product No.: S817881) was obtained from Macklin (Shanghai, China). 3-(4,5-Dimethyl-2-Thiazolyl)-2,5-Diphenyl-2-*H*-Tetrazolium bromide (MTT, Cat. No.: ADB45956003), fluorescein isothiocyanate isomer I (FITC, Cat. No.: AL49003001), dimethyl sulfoxide (DMSO, Cat. No.: ADB75927004), amiloride (Cat. No.: ADB102605001), genistein (Cat. No.: ADB57951002), 4′,6-Diamidino-2-Phenylindole (DAPI, Cat. No.: AL44946001), lyso-detector (Cat. No.: C8085), Dulbecco’s Modified Eagle Medium (DMEM, Cat. No.: C8013), and fetal bovine serum (FBS, Cat. No.: C8010) were purchased from Adamas (Shanghai, China). Penicillin–streptomycin (G4003) was obtained from Servicebio (Wuhan, China). Chlorpromazine (C2481) was purchased from TCI (Shanghai, China). Ethidium bromide (EB, D-9436) was sourced from Bioss (Beijing, China). The 293T cells (Human Renal Tubular Epithelial Cells) were from the National Collection of Authenticated Cell Cultures (Shanghai, China).

### 2.2. Preparation of DNA Complexes

The pre-prepared PEI solution was formulated with a concentration of 150 µg/mL, while the DNA (Salmon sperm DNA or GFP) solution was prepared at a concentration of 100 μg/mL. To facilitate sample preparation, the volumes of both substances were fixed, and their concentrations were adjusted to achieve the desired 1.5:1 (PEI/DNA, *w*/*w*) ratio. The formation of the PEI/DNA complex involved the gradual addition of 100 μL of DNA solution to an equal volume of PEI solution, followed by a 20 min incubation period to facilitate a thorough reaction. Subsequently, the PEI/DNA/Spermine complex was achieved by introducing spermine into the pre-reacted PEI/DNA mixture. The PEI/Spermine/DNA complex was formed by initially combining PEI with spermine for 10 min, followed by dropwise addition of DNA with vigorous stirring, and allowing the reaction to proceed for 20 min.

### 2.3. Particle Size and Zeta Potential Measurement

The hydrodynamic particle size and zeta potential of PEI/DNA, PEI/Spermine/DNA, and PEI/DNA/Spermine were determined by dynamic light scattering (DLS) (ZS90, Malvern Instrument Co., Ltd., Malvern, UK) at 25 °C. Each measurement was conducted in triplicate. The concentration of the above complexes is determined to be 10 μg/mL in water [30].

### 2.4. 293T Cell Culture

293T cells were cultured in Dulbecco’s Modified Eagle Medium supplemented with 10% FBS and 1% penicillin/streptomycin. The cells were incubated at 37 °C in a CO_2_ incubator with 5% CO_2_ for maintenance.

### 2.5. Agarose Gel Electrophoresis

Agarose gel electrophoresis was employed to investigate the binding capability of FITC-labeled spermine with PEI/DNA. FITC was dissolved in DMSO at a concentration of 9 mg/mL; spermine was dissolved in deionized water at a concentration of 4 mg/mL. FITC was incorporated into the spermine aqueous solution under stirring conditions; this mixture was stirred in the dark for a duration of 24 h to yield FITC-Spermine [31]. The preparation of PEI/FITC-Spermine/DNA followed previously delineated methodologies. DNA, FITC, FITC-Spermine/DNA, and PEI/FITC-Spermine/DNA were combined with loading buffer and subsequently loaded onto a 1.0 % agarose gel. The gel was subjected to an electric field of 100 V for 35 min in 1×TAE buffer. Ultimately, the DNA bands were visualized under ultraviolet (UV) light, and the position of spermine was determined by observing FITC fluorescence intensity using a fluorescent imaging device (ABL X5, Tanon, Shanghai, China).

### 2.6. Transfection Protocol

293T cells were plated at a density of 120,000 cells per well in a 24-well plate, and after a period of 16–18 h of incubation, the initial culture medium was removed and substituted with a thinned gene complex sample in media devoid of serum. The pDNA concentration used for our transfection is 2 μg/mL. Subsequent to transfection for a duration of 4–6 h, the initial culture medium was withdrawn and substituted with new media encompassing serum and antibiotics. Subsequent to an incubation period of 24–48 h, the gene expression outcomes were observed using fluorescent inverted microscopy (Eclipse Ti2-U, Nikon, Tokyo, Japan), and the quantification of fluorescence was analyzed utilizing a flow cytometer (C6 Plus, BD Accuri Co., Ltd., Shanghai, China).

### 2.7. Cell Cytotoxicity Analysis

293T cells were seeded at a density of 10,000 cells per well in a 96-well plate and cultured for 24 h. The initial medium was then replaced with a serum-free medium containing different concentrations of spermine or PEI/DNA/Spermine, and a DMEM without sample group was set up as a control. In the preliminary research, it was discovered that the incorporation of spermine into the PEI/DNA complex at a concentration of 100 µg/mL significantly enhanced the transfection efficiency. Consequently, when designing the experimental protocol, a concentration of 100 µg/mL was selected as a pivotal experimental parameter, with additional concentrations established both above and below this value for comprehensive evaluation. After co-incubation with the cells for 4–6 h, a fresh medium was added, and the cells were further cultured for 24 h. Subsequently, 20 μL of MTT solution was added to each well and incubated for 4 h. The supernatant was removed, and 150 μL of DMSO solution was added to each well, followed by shaking on a shaker for 10 min. The absorbance was measured at 490 nm using the enzyme labeler (Synergy NEO, BioTek, Winooski, VT, America). The cell viability of untreated cells was considered as 100% for the control group, and the relative cell viability of the complexes was calculated using the following Formula (1):

Cell viability (%) = (OD_sample_ − OD_blank_)/(OD_control_ − OD_blank_) × 100% (1)

### 2.8. Spermine Combination Screening

Since we determined the optimal transfection effect with a PEI/DNA and PEI/DNA/Spermine mass ratio of 1.5:1 and 1.5:1:25 in previous experiments, we used nanoparticles of this ratio in all subsequent transfection and validation experiments. Based on the PEI/Spermine/DNA mass ratio (1.5:25:1), samples were prepared with a final DNA concentration of 0.1 mg/mL in a total volume of 10 µL. The complexes of PEI/GFP, PEI/GFP/Spermine, and PEI/Spermine/GFP were synthesized and employed in transfection assays in accordance with the aforementioned protocol. Furthermore, an alternative protocol entailed a sequential addition strategy, whereby spermine was initially introduced into the cellular milieu to regulate osmotic pressure, succeeded by the introduction of the gene vector PEI/GFP.

### 2.9. Cellular Internalization Process

293T cells were plated in 24-well plates at a density of 100,000 cells per well and cultured until reaching a cellular density of 70–80%. Subsequently, the cells were individually exposed to chlorpromazine (30 μM), amiloride (50 μM), and genistein (50 μg/mL) in media devoid of serum for 1 h, followed by incubation with PEI/EB-DNA or PEI/Spermine/EB-DNA complexes for 4 h. The concentrations and dosages of the chemical inhibitors were objectively selected after referencing pertinent literature and making appropriate adjustments [32]. Following a cumulative 5 h of inhibition, the fluorescence intensities were quantified via a flow cytometer (C6 Plus, BD Accuri Co., Ltd., Shanghai, China).

### 2.10. Cell Transfection Mechanism

293T cells were plated in 24-well dishes at a concentration of 120,000 cells per well and grown until reaching 70–80% confluency. The cells were then exposed to chlorpromazine (30 μM), amiloride (50 μM), and genistein (50 μg/mL) in a serum-free medium for 1 h [33], followed by transfection with PEI/GFP or PEI/Spermine/GFP for 4 h, resulting in a total incubation period of 5 h. Subsequently, after 24–48 h of incubation, fluorescent images were captured using a fluorescence inverted microscope, and fluorescence intensity was measured using a flow cytometer (C6 Plus, BD Accuri Co., Ltd., Shanghai, China).

### 2.11. Capacity for Buffering Protons

The proton-buffering capacity of the carrier polymer was ascertained through acid–base titration experiments, utilizing a pH meter for quantification. First, 8 mg of PEI25k was diluted in a 150 mM NaCl solution to achieve a concentration of 1 mg/mL [34]. The PEI25k-Sper composite was then combined in a mass ratio of 1:25 and prepared as a solution of 1 mg/mL in NaCl (in the subsequent text, spermine will be referred to as Sper for simplification). Employing NaCl as the reference group, the test solutions were adjusted to a pH of 10 using either 1 M NaOH or 0.1 M HCl. Following this, titration was performed with a 0.1 molar HCl solution, adding 50 μL incrementally and recording the pH of the solution once it stabilized, until it reached a pH of 3.

### 2.12. The Capacity to Evade Lysosomal Degradation

The 293T cells were seeded at a density of 120,000 cells per well on sterile-coated chamber slide, and cultured until achieving a cell density of approximately 70%. FITC-PEI was prepared in accordance with the methodology detailed in the referenced literature [35]. Subsequently, FITC-PEI/DNA and FITC-PEI/Spermine/DNA were individually introduced. After transfection for 1 and 6 h, the cells were initially washed with PBS, followed by staining with 50 nM lyso-detector dye for 30 min to mark the lysosomes in live cells. After 1–2 PBS rinses, the cells were fixed with 4% paraformaldehyde for 10–15 min, washed with PBS, stained with DAPI for 10 min to visualize the cell nuclei, and once again rinsed with PBS. The chamber slides were air-dried, and the cells were affixed on glass slides with glycerol, positioning them between the chamber slide and the glass slide. Subsequently, the slides were sealed, and the intracellular localization of FITC-PEI/DNA or FITC-PEI/Spermine/DNA was observed utilizing confocal laser scanning microscopy (CLSM, Ti2, Nikon Eclipse Co., Ltd., Japan).

### 2.13. Molecular Simulation

Molecular dynamics simulations were performed using the GROMACS 2021 software under the AMBER99SB force field as follows: DNA-PEI System: Construct a simulation box using GROMACS and randomly position one DNA molecule and one PEI molecule within the system space utilizing Packmol. Fill the box with water, employing the Simple Point Charge (SPC) method to characterize the water molecules [36]. DNA-PEI-Spermine System: Construct a simulation box using GROMACS and randomly position one DNA molecule, one PEI molecule, and seven spermine molecules within the system space utilizing Packmol. Fill the box with water, utilizing the SPC water model.

After energy minimization of the two systems, we conducted a 100 ps NVT pre-equilibration and a 100 ps NPT pre-equilibration, followed by a 100 ns production simulation. The simulation was executed at a temperature of 298.15 K and a pressure of 1 Bar. Temperature and pressure regulation were managed by the V-rescale and Parrinello–Rahman methods, respectively. The leapfrog algorithm was employed to integrate Newton’s equations of motion in the simulation, with an integration time step of 2 fs. The Particle Mesh Ewald (PME) method was utilized to compute long-range electrostatic interactions [37]. The leapfrog algorithm was also employed to integrate Newton’s equations of motion in the simulation, with an integration time step of 2 fs. Long-range dispersion corrections are applied to the energy and pressure.

### 2.14. Statistical Analysis

All values in this study were presented as mean ± standard deviation (as error bar) with three or more than three replicates. Imaging results are consistent after three repeated experiments. Statistical analysis was performed with an independent-sample t-test and one-way analysis of variance (ANOVA), followed by multiple comparisons between individual groups. *p* < 0.05 was considered significant.

## 3. Results and Discussion

### 3.1. Characterization of DNA Vectors

**Figure 1:**
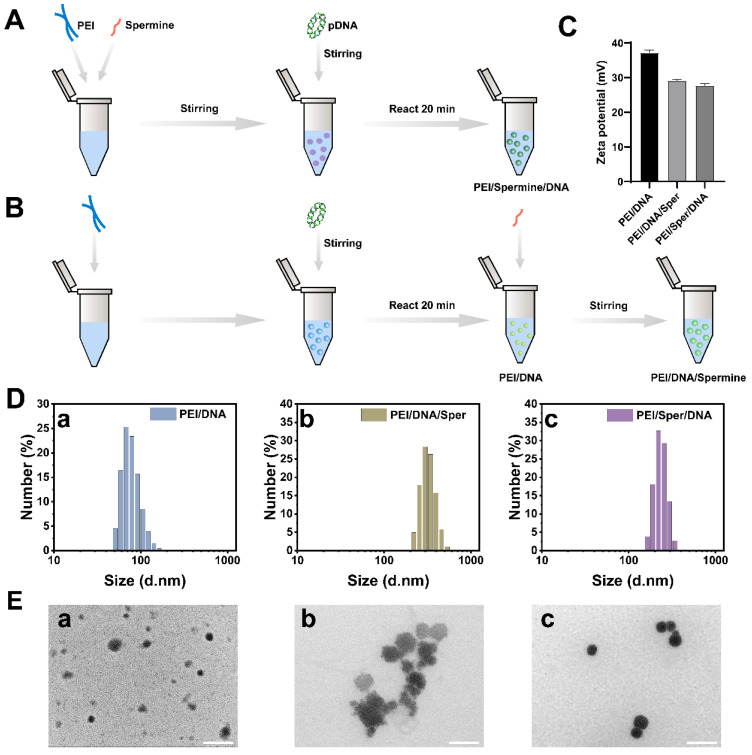
Preparation and characterization of NPs. (**A**,**B**) Synthetic schematic of PEI/DNA/Spermine and PEI/Spermine/DNA. (**C**) Zeta potential of PEI/DNA, PEI/DNA/Spermine, and PEI/Spermine/DNA. (**D**) Particle size distribution of PEI/DNA (**a**), PEI/DNA/Spermine (**b**), and PEI/Spermine/DNA (**c**). (**E**) TEM of PEI/DNA (**a**), PEI/DNA/Spermine (**b**), and PEI/Spermine/DNA (**c**) (scale bar: 200 μm).

The preparation of three complexes was carried out as described previously in Section 2.2. Preparation of DNA Complexes. (Figure 1A,B). The simplest preparation method, physical mixing, was employed to introduce spermine into PEI/DNA. The addition of spermine resulted in a reduction in the surface potential of the system as determined by zeta potential measurements (Figure 1C). The zeta potentials of the complexes prepared with different addition sequences of spermine (PEI/DNA/Spermine and PEI/Spermine/DNA) did not show significant differences, with their surface potentials maintained at approximately +28 mV. Theoretically, the addition of spermine as a cationic substance, to the system should increase the zeta potential, but the results from multiple experiments showed an opposite trend. The most significant factor affecting the zeta potential value was mainly the pH of the solution [38]. Therefore, we measured the pH values of the PEI/DNA solution and the solution after the addition of spermine. The pH of the PEI/DNA solution was 7.50, which increased to 10.15 after the addition of spermine. The pH measurement results indicated that the addition of spermine made the solution environment alkaline, with a larger pH value resulting in a smaller surface potential. Therefore, the addition of spermine changes the pH value of the solution, thereby affecting the surface charge.

We evaluated the hydrodynamic diameter of NPs formed with various sequences of spermine addition and compared them with PEI/DNA complexes lacking spermine (Figure 1D). The hydrodynamic diameter data indicated that PEI/DNA nanocomplexes measured 116.7 ± 7.5 nm. Upon the addition of spermine, the resulting NPs exhibited larger sizes. TEM was employed to characterize the morphology of the nanoparticles. TEM (Figure 1E) and size distribution data (Figure 1D) revealed that PEI/Sper/DNA NPs were approximately 150 nm in size, whereas PEI/DNA/Sper NPs were approximately 200 nm. The final addition of spermine led to the largest DNA vector particles, showing signs of aggregation. In contrast, PEI/Sper/DNA exhibited a more uniform morphology and homogeneous size distribution. Moreover, smaller particle sizes may enhance cellular uptake and facilitate transfection [39,40]. Hence, we hypothesize that PEI/Sper/DNA may offer superior transfection efficiency compared to PEI/DNA/Sper. This result suggests that spermine, as a polyamine, may interact with PEI/DNA, affecting the structure and stability of the complex. Different addition sequences may lead to variations in the binding manner and microstructure among PEI, spermine, and DNA, thereby influencing the properties of the nanoparticles.

### 3.2. Cell Transfection Assay

PEI is a commonly used cationic polymer that is widely applied in the preparation of nanoparticles and gene transfection. As a cationic polymer, PEI can bind to nucleic acid materials (DNA or RNA) with negative charges through electrostatic interactions. As a gene carrier, PEI can help DNA/RNA cross the cell membrane and enter the cytoplasm and nucleus, as well as protect the nucleic acids it carries from degradation by nucleases. 

The ratio of PEI to nucleic acid or the proportion of PEI within the complex significantly influences the efficiency of PEI in delivering genes [41]. The transfection efficacy of PEI/GFP was compared under different mass ratios and concentrations to identify the most favorable transfection conditions. The PEI/DNA ratio of 1.5:1 (*w*/*w*) with a plasmid concentration of 2 µg/mL was ultimately selected due to the balance of the transfection efficiency and cytotoxicity (Figure S1).

**Figure 2:**
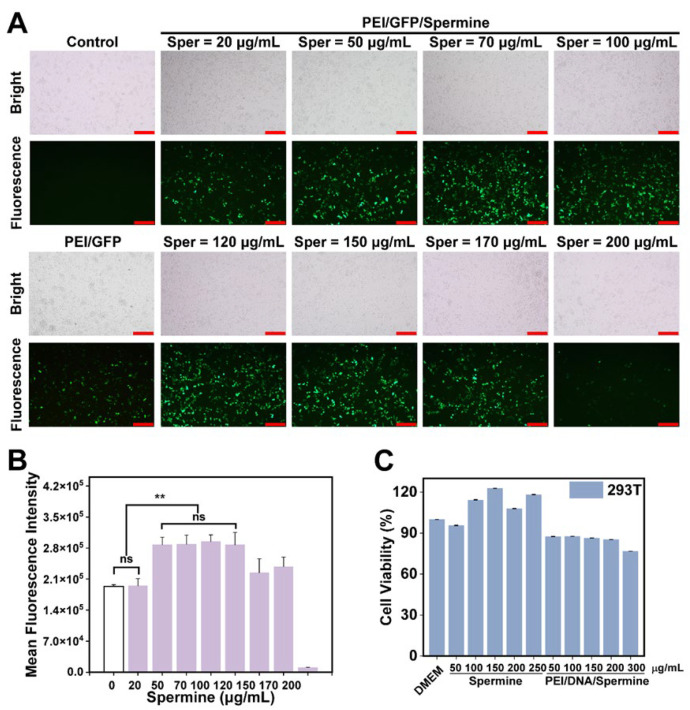
Spermine enhanced in vitro transfection efficiency of PEI. (**A**) The results of 293T cells transfection under an inverted fluorescence microscope. Scale bar is 200 μm. (**B**) Gene transfection efficiency in 293T cells. (ns: *p* > 0.05, ** *p* < 0.01). (**C**) Cytotoxicity test results of different concentrations of spermine and PEI/DNA/Spermine in 293T cells.

Spermine permeates all eukaryotic cells ubiquitously and plays a pivotal role in cellular metabolic processes [42], capable of condensing DNA at specific concentrations. To explore its influence on enhancing PEI carrier transfection efficiency, spermine was integrated into fully reacted PEI/GFP complexes, forming PEI/GFP/Sper NPs. Moreover, the optimal range of spermine concentrations was identified for maximizing transfection efficacy. Through fluorescence microscopy (Figure 2A), a marked enhancement in PEI25k transfection was observed with spermine. Quantitative assessment of green fluorescence emitted from transfected cells via flow cytometry (Figure 2B) indicated that spermine concentrations ranging from 50 to 120 μg/mL notably augmented the transfection capacity of PEI25k. However, excessive spermine (200 μg/mL) diminished transfection efficiency. To mitigate potential cellular impacts, subsequent mechanistic studies employed a concentration of 50 μg/mL spermine. Thus, spermine can work synergistically with PEI to improve transfection efficiency.

PEI forms complexes with nucleic acids through the attraction of its positive charges to the negative charges of the nucleic acids. Spermine, as a polyamine, also carries a positive charge and may bind to DNA via similar electrostatic interactions to form complexes. Furthermore, spermine can condense DNA [27], potentially making the nucleic acid molecules more compact. Concurrently, the synergistic interaction between PEI and spermine on DNA may result in the complete envelopment of the DNA, thereby enhancing its resistance to degradation by intracellular nucleases [43]. Excessively high concentrations of spermine (above 120 μg/mL) can reduce transfection efficiency, possibly due to the over-condensation of nucleic acids into large particles that are difficult for the cell to internalize, or by directly exerting toxicity on the cells.

### 3.3. Cytotoxicity Analysis

Transfection efficacy and cytotoxicity present challenges in gene delivery. Spermine augments PEI-mediated transfection efficacy while preserving minimal cytotoxicity. To evaluate the impact of our vectors and spermine on cell viability, MTT cytotoxicity assays were conducted. The findings (Figure 2C) reveal that spermine concentrations ranging from 50 to 250 μg/mL do not impede cell growth but rather facilitate cell proliferation. Moreover, earlier experiments indicate that PEI/DNA transfection efficacy initially rises with increasing mass ratio, peaking at 1.5:1 before declining. Therefore, using a consistent PEI/DNA mass ratio of 1.5:1, toxicity evaluations were conducted by introducing varying spermine doses. The results demonstrate that our PEI/DNA/Spermine maintains cell viability at approximately 85% with spermine concentrations of 50–200 μg/mL, decreasing slightly to 76% at 300 μg/mL, thus underscoring the relatively low toxicity of our designed vector system.

### 3.4. Transfection with Different Sequences of Spermine

**Figure 3:**
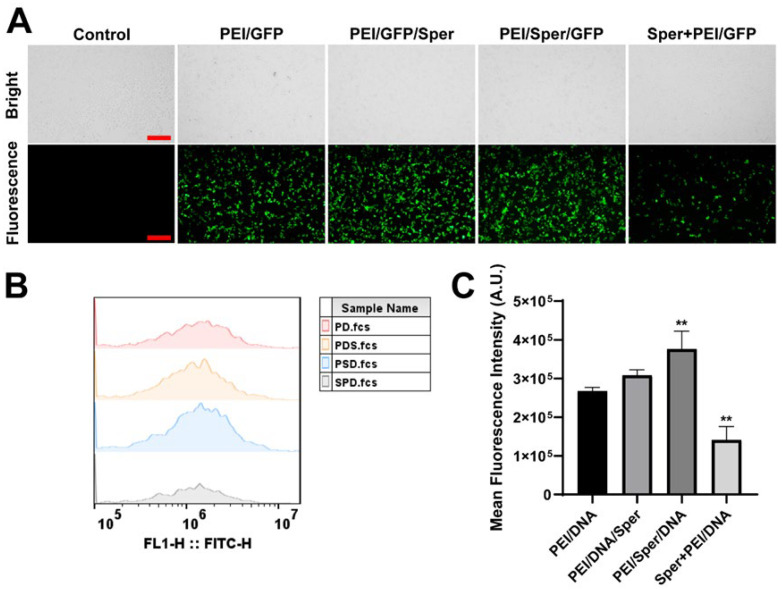
GFP transfection with different spermine addition sequences. (**A**) Fluorescent microscopy images of positive GFP expression in 293T cells treated with different addition spermine sequences. Scale bar is 200 μm. (**B**) Flow cytometry results of gene transfection of NPs in 293T cells. (**C**) Mean fluorescence intensity of positive GFP expression in 293T cells. ** *p* < 0.01.

The impact of the sequence of spermine addition on transfection efficiency was investigated to elucidate the rationale behind the increased efficacy of PEI/GFP transfection. Variations in the order of spermine addition were implemented, resulting in the preparation of PEI/GFP/Sper and PEI/Sper/GFP formulations. Furthermore, recognizing spermine as an alkaline polyamine capable of modifying osmotic pressure upon introduction into the cell culture medium, potentially influencing transfection efficiency in 293T cells [44], a Sper + PEI/GFP group was established. This aimed to precondition the medium with spermine to modulate osmotic pressure prior to PEI/GFP addition, thereby exploring whether the enhancement in transfection efficiency correlates with changes in osmotic pressure. Fluorescence microscopy observations of transfection outcomes (Figure 3A) indicated that spermine and PEI/GFP, when added separately to the cell culture medium, did not enhance PEI/GFP transfection efficiency; rather, they diminished it. This finding suggests that spermine alone does not augment transfection efficiency; effective enhancement occurs only when spermine is combined with PEI/GFP or PEI. Flow cytometry analysis of cells expressing green fluorescent protein revealed that samples prepared by premixing PEI with spermine followed by GFP addition, specifically PEI/Sper/GFP, demonstrated the highest transfection efficiency (Figure 3B,C). This corroborates earlier hypotheses, indicating that the smaller nanoparticle size of PEI/Sper/DNA compared to PEI/DNA/Sper may facilitate cellular transfection. Consequently, this methodology was adopted for subsequent sample preparation and experiments.

The preparation process was optimized by regulating the sequence of spermine addition to achieve optimal transfection efficiency. The experimental results indicate that the approach of premixing PEI with spermine prior to DNA loading significantly enhances transfection efficacy. Consequently, this method was employed exclusively in all subsequent mechanistic investigations for preparing PEI/Sper/DNA. The stability of PEI/Sper/DNA was evaluated by determining the variations in particle size over a span of seven days. Although this straightforward physical mixing does not permit stable storage (as illustrated in Figure S2), the preparation technique is notably simple, requiring on-demand synthesis without the necessity for storage.

### 3.5. The Affinity of Spermine for PEI/pDNA Assemblies

**Figure 4:**
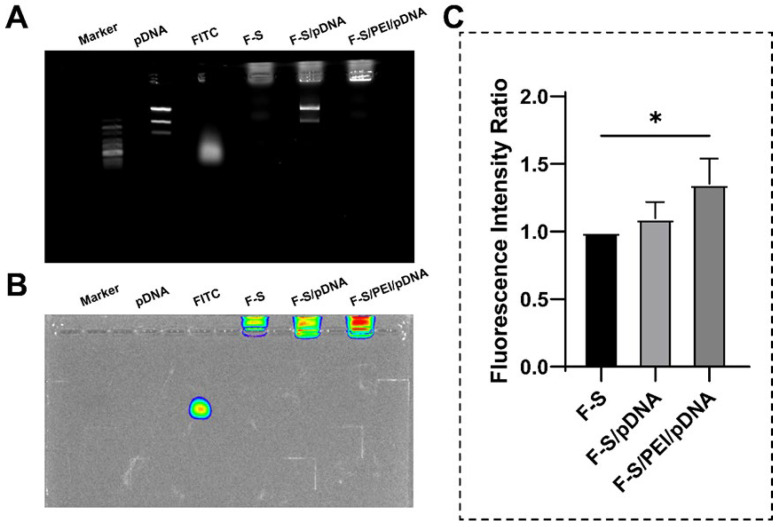
(**A**) Results of agarose gel electrophoresis with pDNA in different complexes. (**B**,**C**) FITC-Spermine binding status in different complexes. * *p* < 0.05.

To investigate whether spermine can form stable complexes with PEI/DNA, agarose gel electrophoresis was employed to observe the band positions of pDNA, and the binding status of spermine within the complexes was determined using a fluorescent imaging system with FITC-labeled spermine. Agarose gel electrophoresis can assess the binding capacity of DNA in different complexes. DNA, possessing numerous phosphate groups and thus a negative charge, migrates freely from the negative to the positive electrode under an electric field. DNA of varying molecular weights migrates at different rates within the gel, with larger molecules moving more slowly. The results from agarose gel electrophoresis revealed that the bands of pDNA alone and pDNA combined with spermine are essentially identical on the agarose gel, with only a negligible amount of DNA confined within the sample wells (Figure 4A). This suggests that spermine alone does not entirely impede the migration of DNA. Additionally, the combination of spermine with PEI/DNA completely restricts the migration of DNA, demonstrating a robust DNA binding capacity.

To further explore the binding capabilities of spermine with PEI/DNA, the fluorescence intensity of FITC-labeled spermine was utilized to locate spermine within the complexes (Figure 4B). Under an electric field, free FITC exhibits fluorescence within the agarose gel. When combined with cationic spermine, FITC, now positively charged, migrates from the sample wells toward the negative electrode region. This outcome indicates successful labeling of spermine with FITC. In comparison to FITC-Spermine, F-S/PEI/pDNA exhibits a significantly stronger fluorescence intensity, whereas F-S/pDNA shows identical fluorescence intensity (Figure 4C). (Furthermore, to ensure the reliability of the results, the amount of F-S in the samples was consistent.) This phenomenon indicates that while spermine cannot bind with DNA, it can bind with PEI/DNA, resulting in a higher concentration of spermine confined within the sample wells and gel, unable to fully migrate beyond the gel. Consequently, spermine is capable of binding with PEI/DNA.

### 3.6. Mechanism of Cellular Uptake

**Figure 5:**
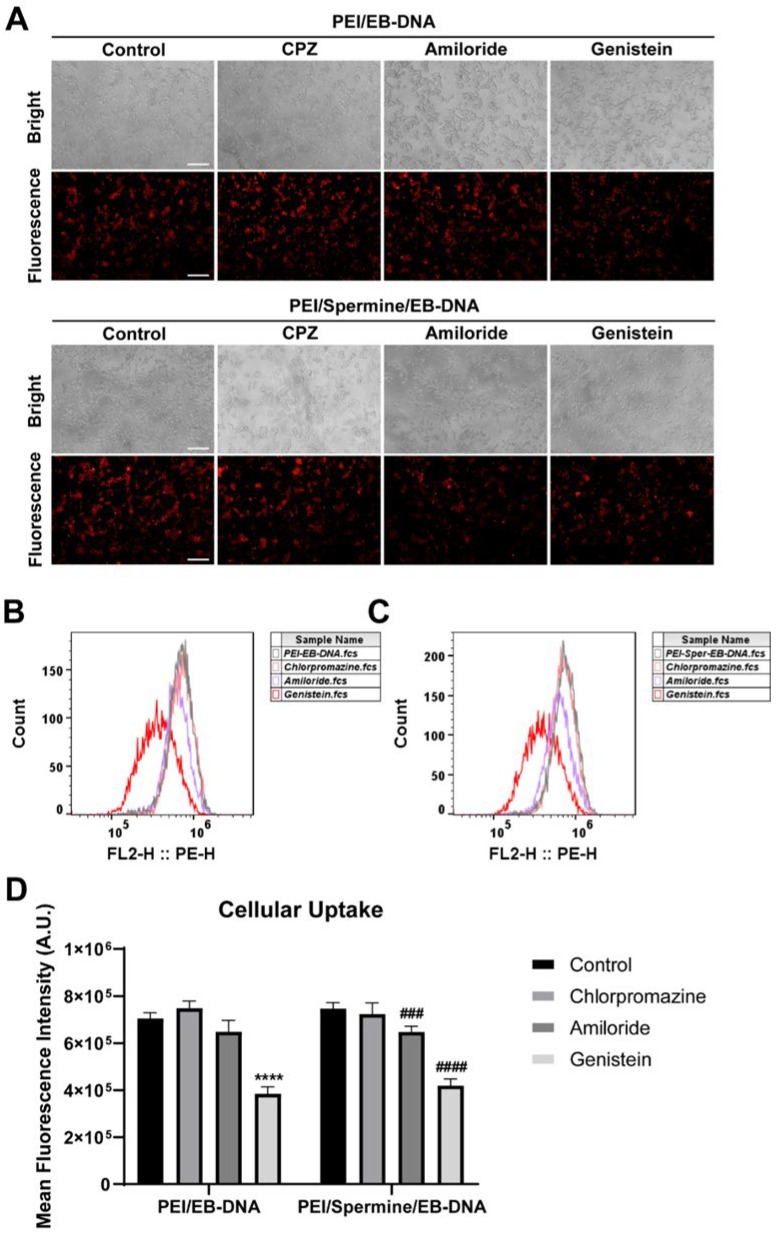
Cellular uptake mechanism study. (**A**) Cellular uptake of 293T cells treated with PEI/EB-DNA NPs and PEI/Spermine/EB-DNA NPs. Scale bar is 200 μm. (**B**,**C**) Flow cytometry results of PEI/EB-DNA (**B**) and PEI/Spermine/EB-DNA (**C**) in 293T cells. (**D**) Mean fluorescence intensity of PEI/EB-DNA (left) and PEI/Spermine/EB-DNA (right) after treatment with different cell uptake inhibitors. **** *p* < 0.0001, vs. control of PEI/EB-DNA; ### *p* < 0.001, #### *p* < 0.0001, vs. control of PEI/Spermine/EB-DNA.

The pathways through which nanoparticles enter cells can be primarily divided into endocytic and non-endocytic pathways. The endocytic pathway can be further categorized into two types: phagocytosis and pinocytosis. Cationic polymers enter cells primarily through endocytic pathways reliant on clathrin, caveolin, and macropinocytosis. The specific uptake route dictates their intracellular destination. Chemical inhibitors suppress particular uptake channels; diminished uptake rates delineate the carriers’ preferred pathway [45]. Chlorpromazine is a cationic amphiphilic drug that can block clathrin-mediated uptake [46]. Amiloride can inhibit macropinocytosis pathway uptake. Genistein can block the caveolin-mediated endocytic pathway. The working concentration of the inhibitors used in this experiment was selected based on literature reports [32]. Analyzing cellular uptake mechanisms pre- and post-spermine introduction clarifies whether spermine modifies uptake pathways and thereby influences transfection efficiency. Quantification of DNA uptake, labeled with ethidium bromide (EB), was conducted. Fluorescence microscopy (Figure 5A) and flow cytometry (Figure 5B,C) provide an objective assessment of uptake results. Experimental findings (Figure 5D) reveal a significant decline in cellular uptake efficiency of nanocomplexes post-genistein inhibition, with reductions of 45% and 44% observed for PEI/EB-DNA and PEI/Sper/EB-DNA, respectively. Amiloride inhibition induces a slight reduction in uptake efficiency for both nanocomplexes, with decreases of 8% and 13% for PEI/EB-DNA and PEI/Sper/EB-DNA, respectively. Genistein notably impedes cellular uptake of both nanocomplexes, indicating predominant entry through caveolin pathways inhibited by genistein, and a minor fraction via amiloride-inhibited macropinocytosis pathways. PEI/Sper/EB-DNA nanocomplexes primarily utilize caveolin pathways inhibited by genistein for uptake. Compared to PEI/EB-DNA, more nanoparticles enter through amiloride-inhibited macropinocytosis pathways.

The experimental findings revealed that cells treated with chlorpromazine exhibited increased uptake, which may be due to the enhancement of uptake efficiency through other uptake channels by chlorpromazine. Similar results have also been reported [47]. In summary, the results of cellular uptake indicate that the caveolin-mediated uptake channel is the primary pathway for both PEI/DNA and PEI/Spermine/DNA nanoparticles. This result also suggests that there is no significant difference in cellular uptake. Therefore, we subsequently investigated the lysosomal escape ability of the two types of nanoparticles within the cell, providing a precise understanding of their transfection mechanism.

### 3.7. Mechanism of Transfection

**Figure 6:**
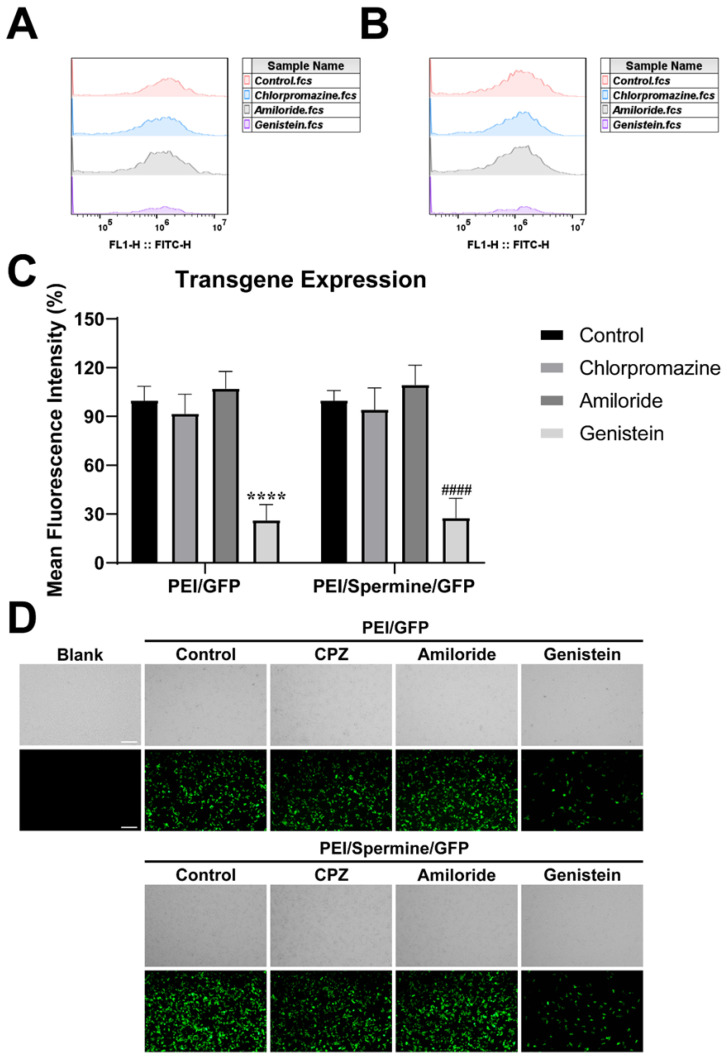
Transgene expression mechanism study. (**A**,**B**) Flow cytometry results of PEI/GFP and PEI/Spermine/GFP after treatment with different inhibitors. (**C**) Mean fluorescence intensity of PEI/GFP (left) and PEI/Sper/GFP (right) after treatment with different cell uptake inhibitors (**** *p* < 0.0001, vs. control of PEI/GFP, #### *p* < 0.0001, vs. control of PEI/Spermine/GFP). (**D**) GFP expression of 293T cells. Scale bar is 200 μm.

Transfection experiments are conducted to validate findings, aiming to mitigate the impact of electrostatic interactions between cationic polymer carriers and cell membranes. Initially, uptake pathways were inhibited using specific inhibitors, followed by the introduction of samples to investigate transfection mechanisms. The results from cell transfection validation (Figure 6A,B) indicate that PEI/Sper/GFP primarily utilizes the caveolin pathway for cellular entry, with a minor fraction entering cells for gene expression via the clathrin pathway, inhibited by chlorpromazine. According to uptake analysis (Figure 6C,D), PEI/Sper/GFP predominantly enters cells through the caveolin pathway, which is hindered by genistein, while the presence of spermine does not significantly alter the gene transfection pathway into cells. However, transfection efficiency notably increases following spermine addition, suggesting that PEI/Sper/GFP enhances transfection efficacy without modifying the uptake pathway to impact transfection. Furthermore, cellular transfection predominantly involves cellular uptake of gene complexes, intracellular delivery, nuclear entry of complexes or genes, translation, and expression. The role of spermine does not influence cellular uptake; thus, its effect on intracellular transport remains a question.

### 3.8. Proton-Buffering Capability of PEI25k-Sper

**Figure 7:**
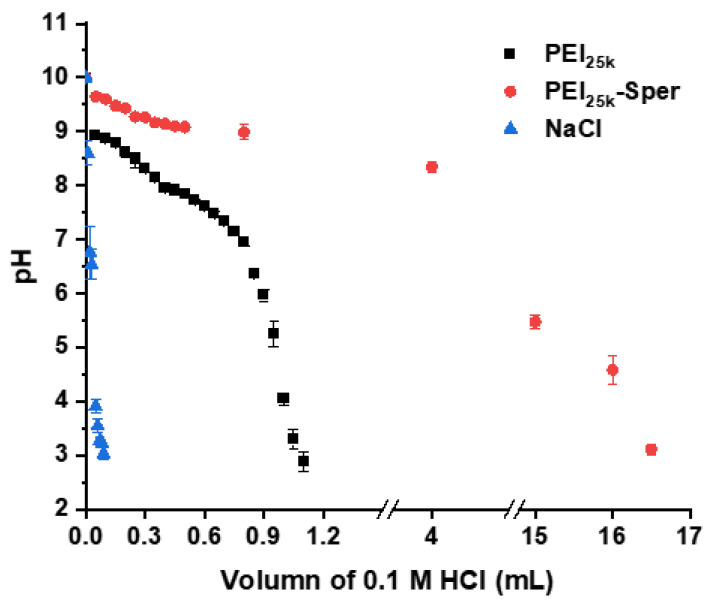
Protonation characteristic of PEI25k and PEI25k-Sper. (Blue, black, and red points symbolize the pH values of NaCl, PEI25k, and PEI25K-Sper, respectively, following the addition of varying volumes of 0.1 M HCl.)

The proton-buffering capacity of carriers was evaluated extracellularly through acid–base titration. The experimental results unveiled a distinct buffering phase for both polymers during titration, highlighting significant buffering capacity (Figure 7). In comparison to the traditional cationic polymer PEI25k, PEI25k-Sper exhibited heightened proton-buffering capacity over a wider pH range. This implies that at physiological pH, PEI25k-Sper binds with nucleic acid anions through electrostatic interactions. At lower pH values, PEI25k-Sper undergoes additional protonation, aiding its escape from endosomes. Hence, the inclusion of spermine enhances the “proton sponge effect” of the original system, facilitating the easier escape of cationic polymers from endosomes for efficient gene release and delivery to the nucleus for expression.

### 3.9. Endosome Escape

**Figure 8:**
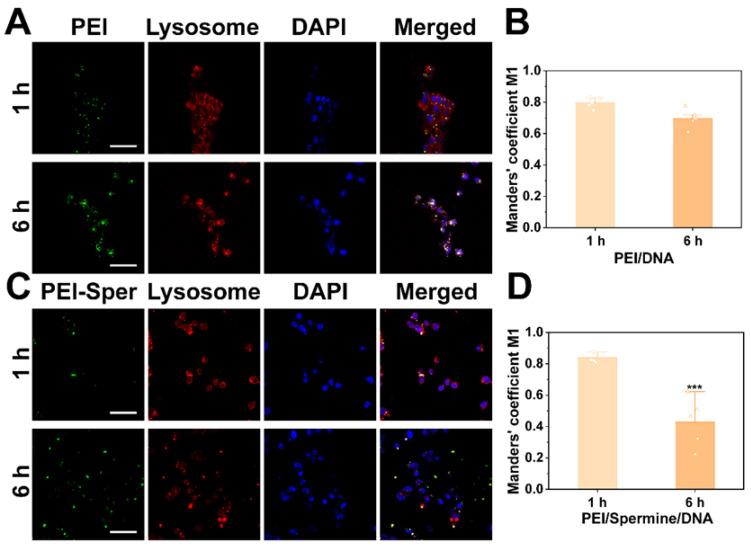
PEI-Sper-mediated nuclear escape assay of DNA. Confocal microscopy images of PEI/DNA nanocomplexes (**A**) and PEI/Spermine/DNA nanoparticles (**C**) transfected into 293T cells for 1 and 6 h. (**B**,**D**) Quantitative analysis of colocalization between FITC-labeled carriers and lyso-detector-labeled lysosomes. Scale bar is 50 μm. *** *p* < 0.001.

The intracellular trafficking of genes can be visualized using confocal laser scanning microscopy (CLSM), confirming the nuclear localization and functional activity of DNA. Lysosomes are fluorescently labeled in red, vectors PEI or PEI-Sper in green FITC, and cell nuclei in blue DAPI to track their intracellular distribution. The orange regions in CLSM data denote intersecting domains between lysosomes and vectors, where a heightened overlap indicates extensive vector presence within lysosomes, thereby indicating ineffective escape. CLSM findings reveal that both PEI and PEI-Sper cohorts are sequestered within lysosomes following 1 h of uptake (Figure 8A,C). After 6 h of uptake, it was clearly observed that nanoparticles from the PEI-Sper group successfully escaped from the lysosomes in 293T cells, while a significant portion of nanoparticles from the PEI group remained confined within the lysosomes. This outcome underscores the superior intracellular release capability of the PEI-Sper vector. The Manders’ coefficient M1 is computed to assess the co-localization of FITC-labeled vectors with red-labeled lysosomes. A coefficient nearing 1 signifies substantial co-localization, while proximity to 0 indicates minimal overlap, thereby signifying superior intracellular release potential. It is evident that following 6 h of nanoparticle uptake, the Manders’ coefficient M1 for the PEI-Sper approximates 0.5, whereas for the PEI, it is around 0.8, accentuating the heightened lysosome escape capability of PEI-Sper (Figure 8B,D).

Based on the previously mentioned uptake pathways and transfection mechanism experimental results, spermine does not affect the cellular uptake of nanoparticles. Additionally, spermine significantly enhances the transfection efficiency of PEI. This enhancement is attributed to spermine increasing the lysosomal escape ability of PEI/Sper/DNA nanoparticles, thereby preventing more nanoparticles from lysosomal degradation and potentially allowing more nanoparticles to enter the cell nucleus for gene expression.

### 3.10. Molecular Dynamics

**Figure 9:**
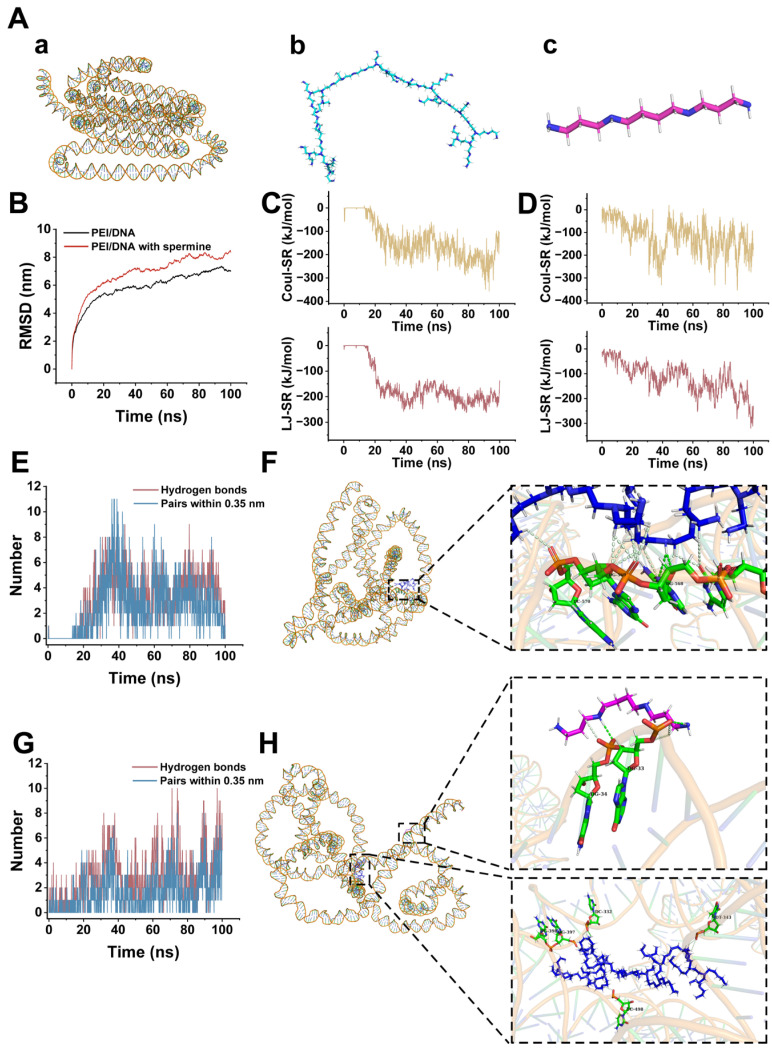
Molecular simulation results. (**A**) Three-dimensional structures of DNA (**a**), PEI (**b**), and spermine molecules (**c**). (**B**) Root Mean Square Deviation (RMSD) curves of the DNA-PEI system and DNA-PEI-Spermine system over time. (**C**) Curves of electrostatic interactions over time for DNA-PEI systems and DNA-PEI-Spermine systems. (**D**) The curves of van der Waals interactions in the DNA-PEI system and the DNA-PEI-Spermine system over time. (**E**) Hydrogen bond quantity curve of DNA-PEI system over time. (**F**) Interaction analysis between DNA and PEI. (**G**) Hydrogen bond number variation curve of DNA-PEI-Spermine system over time. (**H**) Interaction analysis between DNA, PEI, and spermine molecules. The green sticks represent the bases of DNA, blue sticks represent PEI molecules, purple sticks represent spermine molecules, green dashed lines indicate hydrogen bonds, and light green dashed lines indicate carbon–hydrogen bonds.

To further explore the interaction between spermine and the PEI/DNA complex, molecular simulations were employed to analyze the interactions among the three components. We have separately constructed the 3D molecular structures of DNA, PEI, and Spermine (Figure 9A). The RMSD plot (Figure 9B) depicts the RMSD curves of the DNA-PEI system and the DNA-PEI-Spermine system over time. The variation in RMSD over time visually illustrates the structural fluctuations of molecules during the simulation process [48]. An equilibrium or stable state is indicated by a curve that approaches stability, signifying minimal structural changes. The results demonstrate that both systems exhibit convergent RMSD curves with diminishing fluctuations over the simulation duration, validating the stability and reliability of the simulations under the given force field parameters, thus permitting further analysis.

The interaction energy between different molecules or atoms in the system is expressed as the interaction energy. Electrostatic interactions and short-range interactions between molecules are manifested as Coulombic and van der Waals interactions, respectively. Upon completion of the simulation of the DNA-PEI system, the van der Waals energy value was −138.03 kJ/mol, and the electrostatic interaction energy value was −108.56 kJ/mol (Figure 9C). For the DNA-PEI-Spermine system, the van der Waals energy value was −242.49 kJ/mol, and the electrostatic interaction energy value was −169.75 kJ/mol (Figure 9D). Comparison of the van der Waals and electrostatic interactions under both systems reveals that the DNA-PEI-Spermine system exhibits lower van der Waals and Coulombic interactions, indicating that the addition of spermine promotes molecular interactions, leading to a more stable system with lower energy and a more stable structural state (Table 1).

Hydrogen bonds are a common type of interaction force, being stronger than van der Waals forces but weaker than covalent bonds [49]. In nucleic acids, hydrogen bonds mainly exist between base pairs to maintain the stability of the DNA double helix. The hydrogen bonds between genetic vectors and DNA can enhance their interaction, making the formed complex relatively more stable [50]. Hydrogen bonds also play a part in stabilizing the secondary, tertiary, and quaternary structures of nucleic acid molecules. Additionally, we analyzed the changes in hydrogen bond numbers during the simulation, which indirectly reflect the strength of molecular interactions. The DNA-PEI system (Figure 9E) started with zero hydrogen bonds at the beginning of the simulation and ended with one hydrogen bond. The DNA-PEI-spermine system (Figure 9G) saw an increase in hydrogen bonds from zero to five throughout the simulation, suggesting a potential enhancement in molecular interactions.

Finally, we examined the possible interactions in the conformations of both systems. The interaction 3D plot of the DNA-PEI system (Figure 9F) reveals the formation of two sets of hydrogen bonds and eleven sets of weak carbon–hydrogen interactions between the DNA and PEI molecules. The interaction 3D plot of the DNA-PEI-Spermine system (Figure 9H) indicates the formation of three sets of hydrogen bonds and ten sets of weak carbon–hydrogen interactions, with two sets of hydrogen bonds resulting from the interaction between the DNA and spermine molecules. These findings suggest that the incorporation of spermine into the system facilitates the formation of hydrogen bonds, enhancing molecular interactions and contributing to a more stable system, which may elucidate the molecular mechanism underlying the enhancement of transfection efficiency by spermine. More stable nanoparticles may be less likely to be degraded by lysosomes after entering the cell. This could result in more carriers delivering DNA to the nucleus for expression.

## 4. Conclusions

This study employs a simple physical mixing preparation method to combine spermine with PEI as a gene carrier for DNA delivery. The evaluation of PEI/DNA/Spermine and PEI/Spermine/DNA complexes unveiled that the inclusion of spermine augments the nanoparticle dimensions from 116 nm to 150 nm while upholding a more consistent and orderly morphology, as deduced from assessments of particle size, zeta potential, and TEM analysis. By optimizing the addition sequence and amount of spermine, the PEI/Spermine/DNA complex with the optimal transfection efficiency was ultimately determined, thereby establishing a methodology and formulation for their preparation. Toxicological assessments of spermine and its nanocomposites demonstrated favorable safety profiles in vitro. The investigation into the mechanisms of cellular uptake and endosomal escape reveals that the addition of spermine does not alter the pathway of intracellular entry for the gene complexes but rather enhances their ability to escape from endosomes. Molecular simulation results indicate that the incorporation of spermine enhances intermolecular interactions, rendering the molecular bindings more robust and the system more stable, thereby making them less susceptible to degradation by lysosomes. This favorable condition aids in lysosomal escape, thereby facilitating efficient gene expression. Therefore, PEI/Spermine represents a gene vector that is facile to prepare and exhibits superior transfection efficiency. PEI/Spermine is capable of efficiently delivering nucleic acids into the cytoplasm, providing protection against nuclease degradation. However, its application is hindered by its poor biocompatibility, primarily due to its challenging biodegradability and cationic nature. These shortcomings underscore the necessity for further in-depth exploration into methods to concurrently enhance the biocompatibility and transfection efficacy of cationic polymers such as PEI. The introduction of spermine through simple physical mixing to bind non-covalently with DNA can enhance its transfection efficiency, yet it suffers from the drawback of being unsuitable for long-term storage (Figure S2) and application. Due to time constraints, the impact of spermine on the delivery efficiency of other gene carriers has not been investigated, a subject that perhaps warrants further exploration in subsequent studies.

## Figures and Tables

**Table 1:** Analysis of Intermolecular Forces.

Time	System	Electrostatic Interaction	Van der Waals Force	Number of Hydrogen Bonds
0 ns	DNA-PEI	−0.17	−0.13	0
	DNA-PEI-Spermine	3.11	−13.97	0
100 ns	DNA-PEI	−108.56	−138.03	1
	DNA-PEI-Spermine	−169.75	−242.49	5

## Data Availability

Data are contained within the article and Supplementary Materials.

## Supplementary Materials

The following supporting information can be downloaded at: https://www.mdpi.com/article/10.3390/pharmaceutics17010131/s1, Figure S1: Fluorescence intensity of PEI/GFP transfection at different ratios and concentrations; Figure S2: Changes in the size of PEI/DNA/Spermine nanoparticles over a seven-day period.
