# Supplementary material for: Spermine Significantly Increases the Transfection Efficiency of Cationic Polymeric Gene Vectors
Source: Pharmaceutics. 2025 Jan 17;17(1):131. doi: 10.3390/pharmaceutics17010131 (PMC11768368; doi:10.3390/pharmaceutics17010131)
Supplement: Supplementary file 1 [file pharmaceutics-17-00131-s001.zip › pharmaceutics-3417958-supplementary.pdf]

pharmaceutics

MDPI

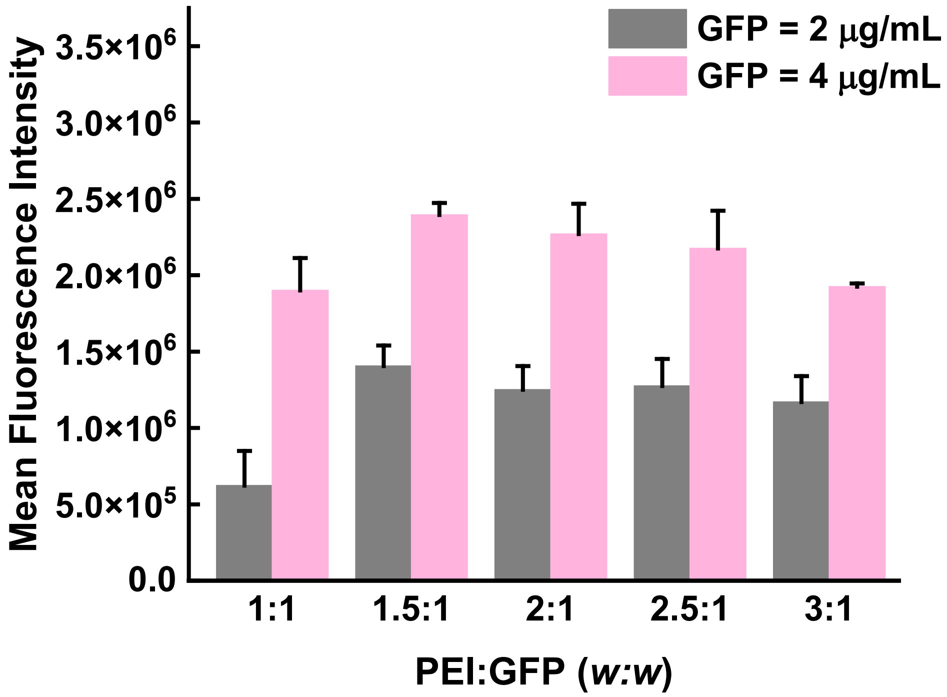

Figure S1. Fluorescence intensity of PEI/GFP transfection at different ratios and concentrations.

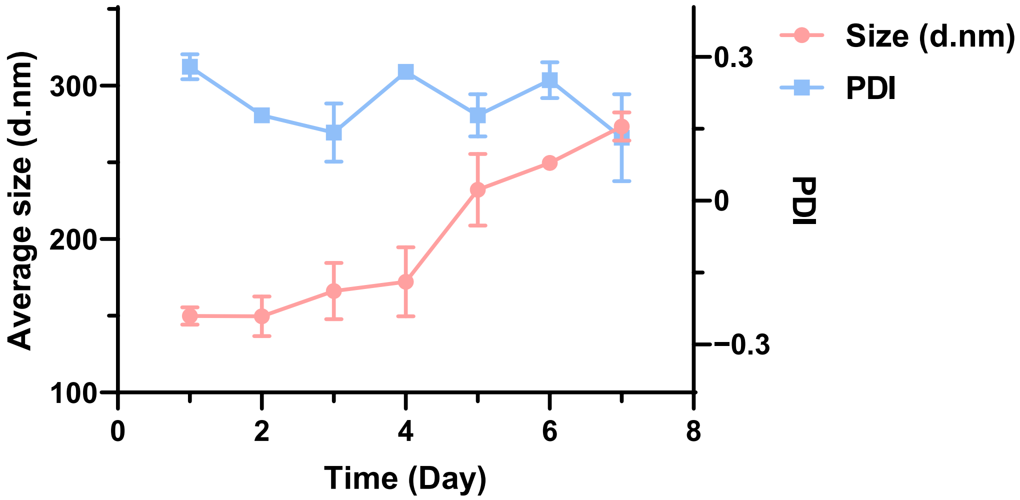

Figure S2. Changes in the size of PEI/DNA/Spermine nanoparticles over a seven-day period. (The blue and red lines respectively denote the trends of PDI and size over time.)
